# Claudin7 and moesin in endometrial Adenocarcinoma; a retrospective study of 265 patients

**DOI:** 10.1186/1756-0500-5-65

**Published:** 2012-01-24

**Authors:** Paulette Mhawech-Fauceglia, Dan Wang, Shashikant Lele, Peter J Frederick, Tanja Pejovic, Song Liu

**Affiliations:** 1 Department of Pathology, Roswell Park Cancer Institute, Buffalo, NY, USA; 2 Department of Biostatistics, Roswell Park Cancer Institute, Buffalo, NY, USA; 3 Department of Gynecologic Oncology at Roswell Park Cancer Institute, Buffalo, NY, USA; 4 Department of Gynecology-Oncology Surgery at Oregon Health and Science, Portland, Oregon, USA; 5 University of Southern California, Los Angeles, CA, USA

**Keywords:** Moesin, Claudin7, Endometrial adenocarcinoma, Clinical outcome

## Abstract

**Background:**

Metastasis is the main cause of death in cancer and is a multistep process. Moesin (MSN), a member of the ezrin-rdixin-moesin family and Claudin7 (CLDN7), a tight junction protein, both play a role in tumor cell metastasis. Previously, we found an over-expression of MSN and under-expression of CLDN7 at the mRNA level in uterine serous carcinoma in comparison to uterine endometrioid adenocarcinoma. The purpose of this study is to determine the protein expression of MSN and CLDN7 in endometrial cancer (EC) and to evaluate their prognostic value. Two hundred sixty-five patients with EC were retrieved from the archives. MSN and CLDN7 immunostaining were performed on the tissue paraffin sections. The expression of each antibody was reported and then correlated with clinicopathological prognostic factors including age, tumor grade, tumor stage, lympho-vascular involvement, depth of myometrial invasion, overall survival (OS), disease free survival (DFS) and death of disease (DOD).

**Results:**

MSN and CLDN were expressed in 46% and 52% of overall cases. We observed an association between MSN*^+ ^*staining and tumor grade, and serous and clear cell carcinoma subtypes (*p *< 0.001 each). There was an association between CLDN7^+ ^staining and low tumor grade and endometrioid adenocarcinoma subtype (*p *< 0.001 and 0.001 respectively). However, no association between MSN and CLDN7 expression and outcome including OS, DOD, and DFS was found.

**Conclusion:**

A significant prognostic value of MSN and CLDN7 in predicting disease outcomes in patients with EC was not demonstrated. Nevertheless, the high percentage of EC cases with MSN and CLDN7 immunoexpression, and their association with tumor grade and subtypes, suggests that these proteins might play a role in tumorigenesis of endometrial adenocarcinomas. Future studies are needed to shed light on their mechanistic properties in EC cells.

## Background

Carcinoma of the endometrium is the most frequently diagnosed malignancy of the female genital tract and is the fifth leading cause of cancer related deaths in women [1]. Endometrial cancer (EC) is divided into two tumor subtypes: type I, a generally less aggressive neoplasm (endometrioid, secretory and mucinous), and type II, a more virulent type (serous, clear cell carcinoma, and undifferentiated carcinoma). Endometrioid adenocarcinomas (EAC) account for more than 80% of cases, and they tend to present as low grade, early stage tumors with favorable outcomes. While uterine serous carcinomas (USC) represent a minority (3-10%) of total endometrial cancer cases, they are usually high grade tumors with deep myometrial invasion, lymphovascular involvement, and a more aggressive clinical course [2-4]. USC is responsible for a disproportionate number of deaths due to the fact that most of these tumors have already spread outside the uteri corpus. In our quest to better understand the clinical behavior of EAC and USC, a DNA microarray was recently conducted and genes of interest were validated by using RT-PCR [5,6]. In this study, two genes involved in tumor cell metastasis were found to be differentially expressed at the mRNA levels in USC in comparison to EAC, with the first (moesin) overexpressed and the second (claudin7) under-expressed.

Metastasis is the primary cause of death and it is a multistep process that requires invasion of the basement membrane by tumor cells, streaming through the blood/lymph vessels and extravasation and growth in distant locations. Moesin or MSN (membrane organizing extension spike protein) is a member of the ERM (ezrin-radixin-moesin) cytoskeleton-associated protein family. This family of proteins acts as a linkage between the cell membrane and the underlying actin cytoskeleton and it has been implicated in maintenance of cell shape, cell motility and membrane trafficking and tumor metastasis [7-11]. EMR proteins share 75% sequence identity and thus it is logical to hypothesize that like ezrin, radixin and moesin might be involved in tumor cell migration [9]. Furthermore, recent studies showed that moesin knock-down increased migration, invasion and metastasis in pancreatic and gastric carcinomas [12,13]. Claudin7 (CLDN7) belongs to the tight junction protein family. This family is composed of 24 proteins and it is critical for maintaining cell polarity and signal transductions. Loss of cell-cell junction is one critical step in tumor cell metastasis [14]. Loss of claudins has been reported in several malignancies and their expression seems to be a prognostic marker in several cancer types [14-16]. More specifically, loss of CLDN7 was reported in breast carcinoma, oral squamous cell carcinoma and colorectal carcinoma where it was found to be associated with poor prognosis in these tumor types [17-19]. The first objective of our study was to explore the MSN and CLDN7 protein expression in a large series of human endometrial cancers. The second objective was to examine the prognostic value of MSN and CLDN7 in predicting disease outcomes in patients with EC. To our knowledge, this is the first report to evaluate MSN and CLDN7 in endometrial cancer patients.

## Methods

### Patients population

The pathology archives were searched for endometrial adenocarcinoma cases from January 2000-December 2010. IRB approval (I-75206) was obtained. A chart review was conducted with extraction of clinical information including the patients' age at the time of diagnosis, the surgical stage, the post-operative therapy, the disease free survival (DFS), the site of recurrence, the cause and the date of death. All patients underwent a surgical staging procedure including a total hysterectomy with bilateral salpingo-oophorectomy, with or without pelvic and para-aortic lymph node dissection and pelvic washings, depending on the tumor grade and the clinical tumor stage. Patients were treated according to the National Comprehensive Cancer Network (NCCN) guidelines (http://www.cancer.gov).

### Histological evaluation

All pathology specimens were reviewed by one pathologist (PMF), and tumors were classified according to World Health Organization (WHO) criteria [20]. All slides were examined by an expert gynecologic pathologist for confirmation of the histologic type, tumor size, tumor grade, depth of myometrial invasion (MI) and presence of lymphovascular invasion (LVI).

### Immunohistochemistry

**Figure 1:**
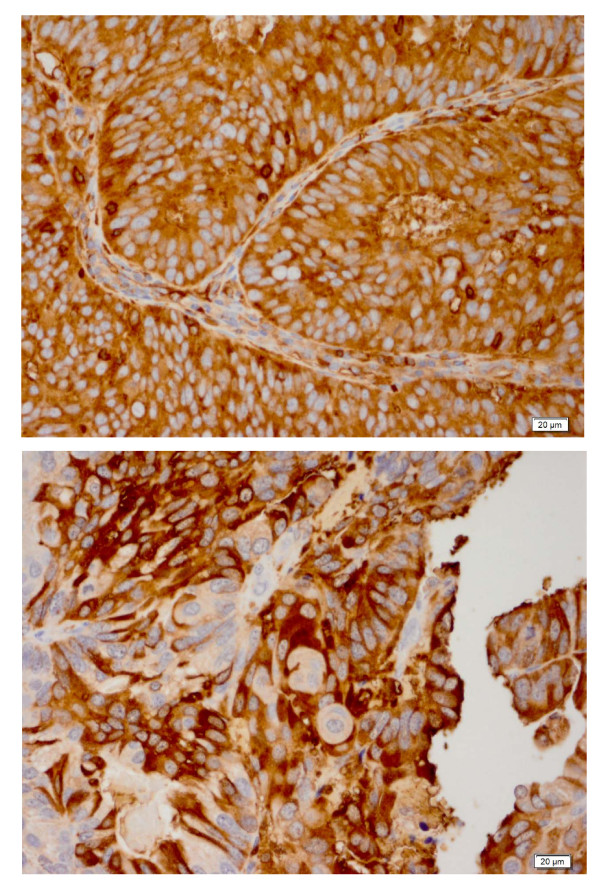
**A Endometrioid adenocarcinoma positive for MSN antibody**. The intensity of the staining is strong and it has a membranous and cytoplasmic pattern. (magnification × 40). Figure 1B: uterine serous carcinoma with positive staining for CLDN7 (magnification x40)

Four μm thick sections were deparaffinized with xylene, and washed with ethanol. Sections were cooled 20 min and incubated 10 min with 3% H_2_O_2 _to quench endogenous peroxidase activity. Blocking was performed using serum-free protein block, Dakocytomation (Carpenteria, CA) for 30 min. The sections were pretreated with an EDTA buffer saline solution, and microwaved for 20 min and then sections were incubated with MSN antibody (monoclonal; 1:20000 dilution; LifeSpan Biosciences, Seattle, WA, USA) and with Claudin7 (polyclonal; 1:50 dilution; Zymed, San Francisco, CA, USA) for 1 h at room temperature. The diaminobenzidine complex was used as a chromogen. Positive control used for MSN was lung adenocarcinoma and for Claudin7 was breast adenocarcinoma. Negative control slides omitting the primary antibody were included in all assays. The stain was membranous and cytoplasmic for both MSN and CLDN7. The extent of immunochemical reactivity was graded based on intensity as follows: 0 (negative), 1+ (weak), 2+ (moderate), 3+ (strong). For the sake of statistical analysis, negative and weak stains were grouped as group I (negative) and moderate and strong as group II (positive). Examples of positive and negative cases are illustrated in Figures 1A-B.

### Statistical analyses

Statistical analyses were performed by R (http://www.r-project.org/). The clinical parameters used for modeling are age, tumor size, histologic subtypes, myometrial depth of invasion, LVI, FIGO grade, recurrence, status, and survival time. To test the association between the biomarker and the clinical parameters, Fisher's exact test was performed for categorical parameters and Welch t- test was used for the continuous ones. For survival analysis, Kaplan-Meier method with log-rank test was used to calculate the cumulative survival time, and check both the overall survival (OS) and disease free survival (DFS) differences between the patients with the different biomarker status. Multivariate cox proportional hazard model was used to determine the hazard ratio that represents the relative risk of death among patients with each of MSN^+ ^and CLDN7^+ ^compared with those with MSN^- ^and CLDN7^-^. All reported p values were two sided.

## Results and discussion

**Figure 2:**
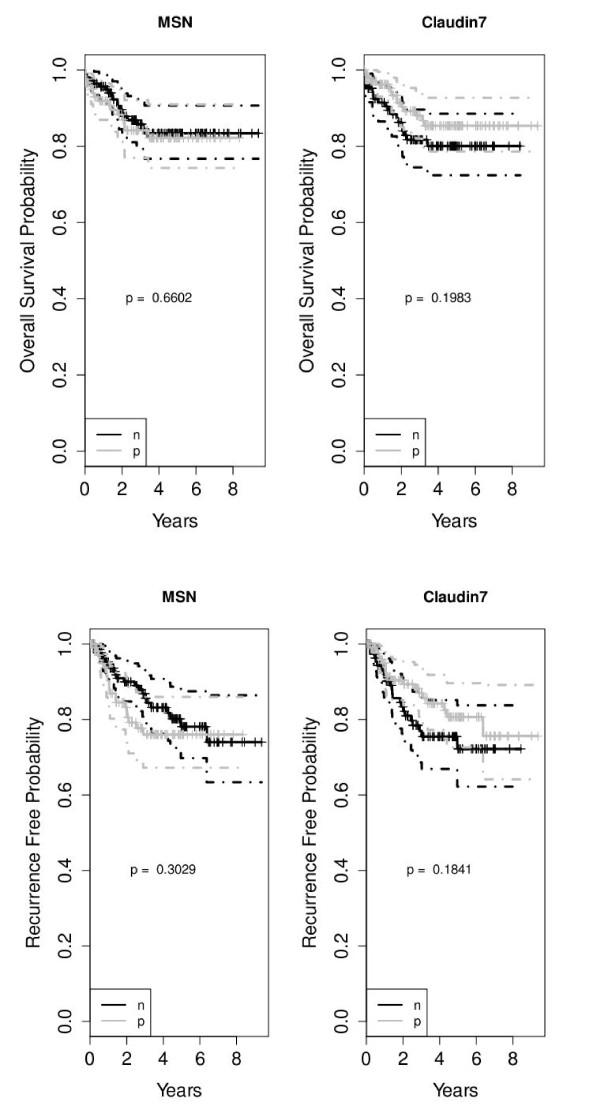
**A Kaplan survival analysis of overall survival probability stratified by MSN/CLDN7 status**. The solid line is the estimated survival for positive (grey) or negative (black). The dash line is the up/lower confidence interval band for positive (grey) or negative (black) B Kaplan survival anlaysis of recurrence probability stratified by MSN/CLDN7 status. The solid line is the estimated survival for positive (grey) or negative (black). The dash line is the up/lower confidence interval band for positive (grey) or negative (black)

The clinical and pathologic features of 265 patients with endometrial adenocarcinoma are summarized in Table 1. All patients had surgery for endometrial cancer with no previous chemotherapy or radiation therapy, and all had complete follow-up information with median of 2.7 years. The distribution of clinical factors in relation to the status of MSN and CLDN7 expression is illustrated in Table 2. There was a strong association between MSN^+ ^and high tumor grade (*p *< 0.001) and with tumor subtype (*p *< 0.001). Specifically, MSN was more likely to be expressed in USC, CCC and carcinosarcoma subtypes, than in endometrioid adenocarcinomas. As for CLDN7, there was a strong association between CLDN7^+ ^and low tumor grade (*p *< 0.001) and endometrioid subtype (*p *= 0.002). There was no association between each of MSN and CLDN7 and disease outcome such as DOD, OS, or DFS (recurrence) (Figure2A, 2B). Finally, MSN and CLDN7 did not show any association with lymph node metastasis.

**Table 1:** Clinical and pathologic features of patients.

**No. of evaluable patients**	265
**Follow time, year**	
**Median**	2.74
**Age, year**	
**Median**	65
**Range**	29-97
**Stage**	
**I**	174(66)
**II**	36(14)
**III**	36(14)
**IV**	19(7)
**Subtype**	
**Endometiriod**	192(73)
**USC+CCC**	54(20)
**Carcinosarcoma**	19(7)
**Grade(FIGO)**	
**1**	113(43)
**2**	49(18)
**3**	103(39)
**Grade(Nuclear)**	
**1**	88(33)
**2**	70(26)
**3**	107(40)
**Tumor size, cm**	
**Median(Range)**	4.58(0-33)
**< = 2**	53(20)
**> 2**	212(80)
**Depth of myometrial invasion**	
**Median(Range)**	38.52(0-100)
**< = 50**	170(64)
**> 50**	95(36)
**Lympho-vascular involvement**	
**no**	192(72)
**yes**	73(28)
**Lymph Node Status**	
**yes**	41(15)
**no**	119(45)
**Not examined**	105(40)
**Recurrence**	
**no**	209(79)
**yes**	42(16)
**persistent**	10(4)
**progression**	4(1)
**Status**	
**ANED**	202(76)
**AWED**	28(11)
**DOD**	22(8)
**DNED**	6(2)
**DWED**	4(2)
**others**	3(1)
**MSN**	
**negative**	143(54)
**positive**	122(46)
**Claudin7**	
**negative**	127(48)
**positive**	138(52)

(Data in parentheses are percentages)

**Table 2:** Association between MSN and claudin7 immunoexpression and the clinical variables

Variables (group 1 vs group2)	MSN			Claudin7		
	*P*value	Odds Ratio	CI	*P*value	Odds Ratio	CI
Age*	0.206			0.196		
Stage^	0.29	1.401	0.741-2.664	1	1.033	0.546-1.962
Tumor size^	0.013	2.305	1.168-4.724	0.759	0.876	0.456-1.673
Lymho-vascular involvement^	0.098	0.616	0.344-1.096	0.413	1.255	0.707-2.236
Lymph node status	1	1.018	0.47-2.2	0.365	0.685	0.314-1.48
Depth of myometrial invasion^	0.305	1.323	0.776-2.262	0.442	0.797	0.467-1.358
Grade_FIGO^	< 0.001	2.561	1.501-4.41	< 0.001	0.389	0.226-0.665
Grade_nuclear^	< 0.001	2.557	1.504-4.385	0.001	0.419	0.245-0.711
Subtype#	< 0.001	0.311	0.154-0.609	0.002	2.628	1.353-5.253
Recurrence#	0.867	0.935	0.456-1.925	0.309	1.479	0.722-3.061
Recurrence^	0.867	0.928	0.454-1.903	0.401	1.383	0.678-2.852
Status#	0.567	0.847	0.462-1.554	0.112	1.623	0.885-3.007
Status^	1	1.015	0.468-2.226	0.278	1.53	0.705-3.386

^ The *P*-values by Fisher exact test to test the correlations between the biomarker and the clinical factors.

* The *P*-value is calculated by the two sample welch t-test

Stage: Group1: stages I and II; Group 2: stages III and IV

Tumor Size: Group 1:< = 2 cm; Group2: > 2 cm

LVI: Group 1:yes; Group2: no

Depth of myometrial invasion: Group1:< = 50; Group2:> 50

Grade: Group1: grade 1 and 2; Group2; grade 3

Subtype#: Group1:CCC & USC; Group 2: Endometrioid; Group3: MMMT

Recurrence#: Group1:yes; Group2:no; Group3:others

Recurrence^: Group1:yes; Group2: no &others

Status#: Group1: ANED; Group2: all others

Status^: Group1:ANED&AWED; Group2: all others

Interprete the Odds Ratio:

> 1, the proportion of p and n biomarkers status in Group 2 is greater than the proportion in Group 1

< 1, the proportion of p and n biomarkers status in Group 1 is greater than the proportion in Group 2

We further explored the impact of MSN and CLDN7 on chemotherapy response. Of the 72/265 patients who received radiation + chemotherapy or chemotherapy alone, 39 were MSN^+ ^while 33 were MSN^-^, and 33 were CLDN7^+ ^while 39 were CLDN^-^. Based on our analysis, there was no significant independent value of MSN and CLDN7 in predicting OS, DFS, or DOD between these two groups (data not shown).

Metastasis is the primary cause of fatality in endometrial cancer. The ERM and claudins are two families of proteins involved in the multistep tumor metastasis process[8,9,14]. Much is known regarding ezrin and claudins1-4 in endometrial cancer but the role of MSN and CLDN7 is yet to be explored [15,21-24]. Based on previous DNA microarray analysis, we showed an up-regulation of the *MSN *gene and a down-regulation of the *CLDN7 *gene in USC in comparison to EAC. Their mRNA expressions were validated by qRT-PCR [5,6]. In addition, MSN was under-expressed and CLDN7 normally expressed in HEC1A and RL95-2 cell lines (data not shown). The present study was done in continuity to our previous work, aiming to determine the protein expression of each of MSN and CLDN7 in EC and to explore their prognostic significance in a large series of patients.

Ezrin is the only gene in the ERM family that has been widely explored in various malignancies. Ezrin was evaluated in EC where it was implicated in the process of invasion in endometrial cancer cell lines [25]. In addition, strong ezrin immunoexpression was related to poor prognosis in FIGO stage I EAC [26]. On the other hand, MSN is less well studied in malignancies and the few published data showed that alteration of MSN was present in pancreatic cancer, lung adenocarcinoma and oral squamous cell carcinoma [12,27,28]. To the best of our knowledge, MSN expression has never been reported in human EC. Previous studies showed that claudin 3 and 4 were overexpressed in USC and they were associated with higher tumor grade [15,23]. However, these studies failed to show an independent value for claudin3 and 4 in predicting disease outcome. In our current investigation, we showed that MSN and CLDN7 proteins were expressed in a high percentage (almost 50%) of EC cases. Furthermore, we found a strong association of MSN and CLDN7 expressions with two important histologic prognostic factors - tumor grade and tumor subtypes. Specifically, MSN protein expression was associated with type II carcinomas and high tumor grade (G3), while CLDN7 protein expression was associated with low tumor grade (FIGO G1 and G2) and with type I carcinomas. Even though no prognostic value of MSN and CLDN7 expression in predicting EC patient outcome was found, the above data nevertheless led us to suggest that MSN and CLDN7 proteins might be involved in the tumorigenesis of endometrial cancer.

One limitation of our study was that the majority of our tumors were endometrioid type, well differentiated, and presented at an early stage, which is a frequent occurrence in endometrial cancer. Because these tumors have favorable outcome in general, it is expected that the majority of the patient population will be alive at the time of last follow-up. The resulting fewer numbers of unfavorable outcome in these patients might limit our statistical power in predicting survivals.

## Conclusions

Using immunohistochemical stains, our work is the first to comprehensively study the protein expression of MSN and CLDN7 in correlation with the clinical characteristics in a large series of patients with endometrial cancer. Although we did not find a significant prognostic value of MSN and CLDN7 in predicting disease outcome in patients with endometrial cancer, our data suggests that MSN and CLDN7 protein immunoexpression might be involved in the development and progression of carcinoma of the endometrium.

## Competing interests

The authors declare that they have no competing interests.

## Authors' contributions

PMF wrote the manuscript; DW and SL performed the statistical analysis and revised the manuscript; TP provided critical insights; PJF and SL collected the data, follow-up, revised the manuscript and provided critical insights of the content of the manuscript. All authors read and approved the final manuscript.
